# Fibroblast activation protein targeted near infrared photoimmunotherapy (NIR PIT) overcomes therapeutic resistance in human esophageal cancer

**DOI:** 10.1038/s41598-021-81465-4

**Published:** 2021-01-18

**Authors:** Ryoichi Katsube, Kazuhiro Noma, Toshiaki Ohara, Noriyuki Nishiwaki, Teruki Kobayashi, Satoshi Komoto, Hiroaki Sato, Hajime Kashima, Takuya Kato, Satoru Kikuchi, Hiroshi Tazawa, Shunsuke Kagawa, Yasuhiro Shirakawa, Hisataka Kobayashi, Toshiyoshi Fujiwara

**Affiliations:** 1 Department of Gastroenterological Surgery, Okayama University Graduate School of Medicine, Dentistry, and Pharmaceutical Sciences, 2-5-1 Shikata-cho, Kita-ku, Okayama, 700-8558 Japan; 2 Department of Pathology and Experimental Medicine, Okayama University Graduate School of Medicine, Dentistry and Pharmaceutical Sciences, Okayama, Japan; 3 Center for Innovative Clinical Medicine, Okayama University Hospital, Okayama, Japan; 4 Molecular Imaging Program, Center for Cancer Research, National Cancer Institute, US National Institutes of Health, Bethesda, MD USA

**Keywords:** Cancer microenvironment, Cancer therapeutic resistance, Chemotherapy, Targeted therapies

## Abstract

Cancer-associated fibroblasts (CAFs) have an important role in the tumor microenvironment. CAFs have the multifunctionality which strongly support cancer progression and the acquisition of therapeutic resistance by cancer cells. Near-infrared photoimmunotherapy (NIR-PIT) is a novel cancer treatment that uses a highly selective monoclonal antibody (mAb)-photosensitizer conjugate. We developed fibroblast activation protein (FAP)-targeted NIR-PIT, in which IR700 was conjugated to a FAP-specific antibody to target CAFs (CAFs-targeted NIR-PIT: CAFs-PIT). Thus, we hypothesized that the control of CAFs could overcome the resistance to conventional chemotherapy in esophageal cancer (EC). In this study, we evaluated whether EC cell acquisition of stronger malignant characteristics and refractoriness to chemoradiotherapy are mediated by CAFs. Next, we assessed whether the resistance could be rescued by eliminating CAF stimulation by CAFs-PIT in vitro and in vivo. Cancer cells acquired chemoradiotherapy resistance via CAF stimulation in vitro and 5-fluorouracil (FU) resistance in CAF-coinoculated tumor models in vivo. CAF stimulation promoted the migration/invasion of cancer cells and a stem-like phenotype in vitro, which were rescued by elimination of CAF stimulation. CAFs-PIT had a highly selective effect on CAFs in vitro. Finally, CAF elimination by CAFs-PIT in vivo demonstrated that the combination of 5-FU and NIR-PIT succeeded in producing 70.9% tumor reduction, while 5-FU alone achieved only 13.3% reduction, suggesting the recovery of 5-FU sensitivity in CAF-rich tumors. In conclusion, CAFs-PIT could overcome therapeutic resistance via CAF elimination. The combined use of novel targeted CAFs-PIT with conventional anticancer treatments can be expected to provide a more effective and sensible treatment strategy.

## Introduction

Stromal cells are strongly involved in the progression of cancer cells, and among stromal cells, cancer-associated fibroblasts (CAFs) have been reported to have an important role in the tumor microenvironment. In the past, several studies have demonstrated the multifunctional potential of CAFs, as they profoundly support cancer growth, invasion, metastasis and acquisition of therapeutic resistance in cancer cells^1–4^. Of note, recent studies have shown the immunosuppressive effects of CAFs on the immune response^5,6^, and we also reported that CAFs regulated immunosuppressive tumor-infiltrating lymphocyte populations via interleukin (IL)-6 in the tumor microenvironment^7^.

Esophageal cancer (EC) is known to be an aggressive malignant tumor. Although many treatment modalities for EC have been developed^8^, the Comprehensive Registry of Esophageal Cancer in Japan has reported that the 5-year survival rates of patients treated using esophagectomy with concurrent chemotherapy, radiotherapy alone or chemoradiotherapy were 6.3%, 24.9% and 32.4%, respectively, which are not satisfactory outcomes and are in part considered to be due to tumor acquisition of therapeutic resistance^9^. Various mechanisms have been proposed, and some studies have indicated the possible involvement of CAFs^10–13^. Thus, overcoming therapeutic resistance is one of the challenges in our efforts, and the development of novel therapeutic approaches is needed to improve the prognosis of EC patients.

Concerning therapeutic resistance associated with the tumor stroma, some studies have already reported that CAFs promote epithelial–mesenchymal transition (EMT) and stem-like characteristics in cancer cells by secreting soluble factors, such as transforming growth factor-beta (TGF-β) and IL-6^14–17^; these phenotypes can be associated with therapeutic resistance, and recently, therapy-induced DNA damage-induced secretion by surrounding fibroblasts^18,19^ and CAF-derived exosomes^20,21^ were also shown to strongly contribute to resistant tumors^22–24^. Furthermore, it has been reported that not only such indirect factors but also direct influences, such as cell-to-cell contact^25,26^ and the increased internal pressure of a CAF-rich stroma, contribute to resistance. In this way, CAFs support tumor acquisition of therapeutic resistance and tumor growth. Thus, many types of therapeutics targeting stromal cells have been investigated; however, CAFs have not yet been established as sufficient treatment targets.

CAFs express several markers, such as α-smooth muscle actin (αSMA), fibroblast stimulating protein-1 (FSP-1), platelet-derived growth factor (PDGFR) α, and PDGFRβ^2,27,28^. Of those markers, we identified a specific surface marker, fibroblast activation protein (FAP), to target CAFs. FAP is a type II cell surface-bound transmembrane glycoprotein^29^ expressed on the cell surface of activated fibroblasts in the reactive stroma of epithelial cancers and the granulation tissue of healing wounds^30^. Interestingly, FAP is expressed in the stroma of more than 90% of human cancers^31^ and has an important role in tumor progression^32^, with high FAP tumor expression being associated with a poor prognosis^33–35^. Our previous reports also demonstrated that FAP-positive cells were strongly related to lymph node metastasis and correlated with shortened survival in EC^36^. Thus, based on those reports, the target molecule FAP is considered to be the most effective marker due to its specificity and malignant relations, even though CAFs express several markers and exhibit heterogeneity.

Near-infrared photoimmunotherapy (NIR-PIT), which was introduced by Mitsunaga et al.^37^ in 2011, is a novel cancer treatment that uses a highly selective monoclonal antibody (mAb)-photosensitizer conjugate (APC). NIR-PIT is a molecularly targeted phototherapy for specific cells based on injecting a conjugate of IRdye700DX (IR700, silicaphthalocyanine dye) and a monoclonal antibody that recognizes an expressed antigen on the target cell surface. Once the antibody-IR700 conjugate is bound to its target, subsequent local exposure to NIR light causes physical changes in the shape of antibody antigen complexes that are thought to induce physical stress within the cellular membrane leading to increases in transmembrane water flow that eventually lead to cell bursting, also known as an immunogenic cell death (ICD), in contrast to most other treatments that result in apoptosis. ICD rapidly mature dendritic cells adjacent to dying cancer cells, resulting in re-education and subsequent proliferation of CD8 + T cells against a variety of released cancer antigens, which amplifies the therapeutic effect of NIR-PIT^38,39^. NIR-PIT is quite different from the conventional photodynamic therapy (PDT), which uses porphyrin derivatives to produce reactive oxygen species in the cells to induce apoptosis and cytotoxic effect^40^. Although recent studies have shown that PDT also induces ICD limited to a few photosensitizers^41^, the induction of host immunity through ICD is what makes NIR-PIT not only a local therapy but also a systemic therapy and is the advantage of NIR-PIT over PDT.

NIR-PIT and PDT are similar in that they induce cytotoxicity by irradiating light on photosensitizers that have accumulated in the tumor. However, unlike PDT, which allows some accumulation of photosensitizers in normal tissues, NIR-PIT selectively binds to specific antigens, leading to selective killing of target cells without destroying the surrounding cells. There are already several reports on NIR-PIT targeting various cells, including epithelial cancer cells and immune cells^42–46^. We also have been attracted to NIR-PIT as a CAFs-specific targeted therapy and developed FAP-targeted NIR-PIT, in which IR700 was conjugated to a FAP-specific antibody to target FAP positive cells. Recently, we successfully demonstrated a promising inhibitory effect on CAFs in EC^47^. Given the above information, as the next challenge of CAFs-targeted NIR-PIT (CAFs-PIT), we hypothesized that the elimination of CAFs could overcome the resistance to chemoradiotherapy described above.

In this work, we aimed to verify that CAFs-PIT could overcome the resistance to chemoradiotherapy in EC. First, we assessed whether EC cells acquire stronger malignant characteristics under the influence of CAFs and are refractory to chemotherapy and radiotherapy. Next, we determined whether the treatment resistance of these carcinoma cells could be rescued under normal conditions without CAF stimulation in vitro, and finally, we evaluated whether therapeutic sensitivity could be improved by CAFs-PIT therapy in vivo*.*

## Results

### CAFs drive resistance to conventional therapy in tumor cells

**Figure 1:**
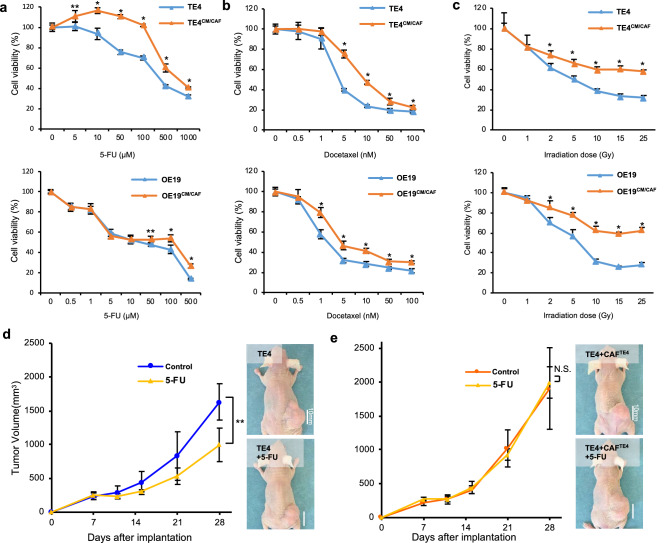
Tumor cells stimulated with CAFs were relatively refractory to conventional therapy in vitro and in vivo. (**a**,**b**) After 48 h of stimulation with CAF conditioned medium, tumor cells were treated with the indicated concentrations of 5-FU (**a**) and docetaxel (**b**), and cell viability was measured. Stimulated tumor cells (the TE4^CM/CAF^ or OE19^CM/CAF^ group) were more resistant to chemotherapy than unstimulated cells. (**c**) Stimulated tumor cells were irradiated with the indicated dose of radiotherapy, and cell viability was measured. Stimulated tumor cells were more resistant to radiotherapy than unstimulated cells. (**d**) The treatment resistance of coinoculated tumors (CAFs and tumor cells) in vivo was evaluated. After inoculation, tumor growth was monitored from day 7 to day 28. (**d**) In the TE4 cells alone group (n = 4), 5-FU suppressed tumor growth in TE4 subcutaneous tumors. (**e**) In the group of mice inoculated with TE4 cells cocultured with CAFs (n = 4), tumors acquired chemoresistance to 5-FU (scale bar, 10 mm). Data are shown as the mean ± SD of three or more independent experiments. Statistical analyses were performed using Student’s t test. *P < 0.05, **P < 0.01. N.S. indicates no significant difference.

To evaluate the influence of CAFs on the therapeutic resistance of cancer cells, we performed a cell viability assay with tumor cells stimulated by CAFs. First, we analyzed the resistance to chemotherapy (5-fluoreuracil (5-FU) or docetaxel). After 2 days of stimulation with conditioned medium (CM) from CAFs (CM/CAF^TE4^ or CM/CAF^OE19^), TE4 and OE19 cells demonstrated more resistance to chemotherapy than unstimulated control cells (Fig. 1a,b). Next, we further analyzed the resistance to radiotherapy. As a result of chemotherapy, stimulated tumor cells demonstrated increased resistance to radiotherapy (Fig. 1c). However, a tendency toward acquired resistance was not demonstrated in cancer cells stimulated with FEF3 cells (CM/NF) as normal fibroblasts (Supplementary Fig. 1). From these results, we planned an in vivo study to confirm the resistance to 5-FU, which is a standard anticancer drug for EC. To evaluate the therapeutic resistance of CAFs in vivo, we compared two groups: TE4 cells inoculated alone vs TE4 and CAFs coinoculated. In the TE4 cells alone group, tumor growth could be suppressed by 5-FU compared with control treatment (39.0% reduction, day 28, P < 0.05) (Fig. 1d). However, in the CAF coinoculated group, a significant difference was not observed between the two groups (Fig. 1e), demonstrating acquired resistance to 5-FU in tumor cells cocultured with CAFs. These results indicated that EC tumors can acquire chemoresistance via CAF stimulation during tumor progression.

### CAFs promote a malignant phenotype in tumor cells

**Figure 2:**
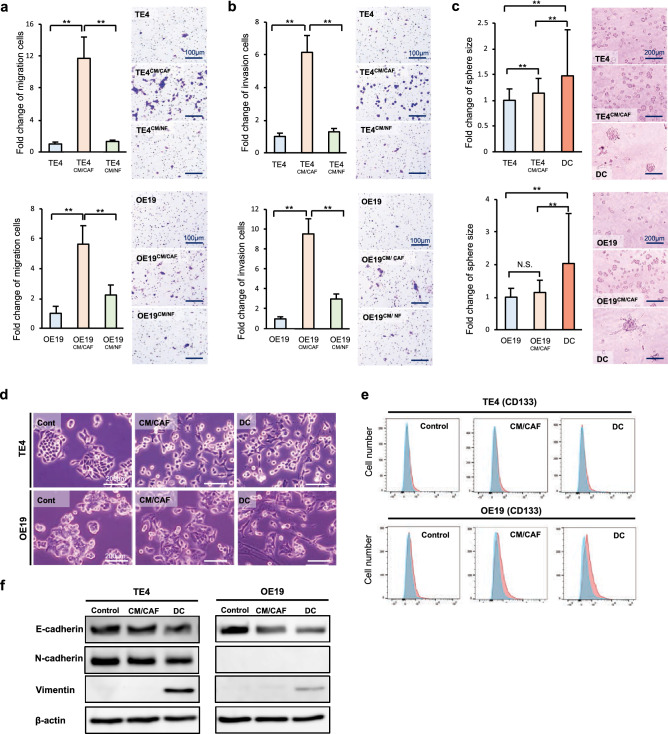
Tumor cells stimulated with CAFs acquired malignant characteristics. The migration (**a**) and invasion (**b**) of esophageal cancer cells cultured with conditioned medium from CAFs were observed and quantified. CAFs enhanced the migration and invasion of cancer cells (scale bar, 100 μm). (**c**) The results of a spheroid formation assay are shown. Spheroid formation was enhanced by CAFs. The spheroid sizes in the direct-contact (DC) groups were significantly larger than those in the control groups (scale bar, 200 μm). (d) The morphological changes of cancer cells stimulated with CAFs are shown. Compared with untreated control cells, the stimulated tumor cells had a larger population with fewer cell junctions and elongated pseudopodia. These changes were observed more clearly with direct contact between the cancer cells and CAFs (scale bar, 200 μm). (**e**) The population of CD133-positive cells was analyzed by flow cytometry. A significant difference was not observed in TE4 cells. However, OE19 cells stimulated with CAFs contained more CD133-positive cancer stem-like cells than unstimulated OE19 cells. (**f**) WB demonstrated that E-cadherin expression was decreased and vimentin expression was increased in CAF-stimulated tumor cells. These changes were observed to be strong in cultures with direct contact between the cancer cells and CAFs. Data are shown as the mean ± SD of three or more independent experiments. Statistical analyses were performed using Dunnett test. **P < 0.01. N.S. indicates no significant difference.

To explore the effects of CAFs on cancer cells with malignant potential, we performed migration, invasion and colony formation assays. The migration assay showed that the number of migrated cancer cells was increased in each cancer cell line for tumor cells stimulated with CM from CAFs (CM/CAF^TE4^ or CM/CAF^OE19^) compared with control tumor cells, whereas migration was not upregulated in cancer cells stimulated with normal fibroblasts (CM/NF) (Fig. 2a). The invasion assay also showed that the number of invaded cancer cells was increased by CM/CAFs in the same manner (Fig. 2b). In the scratch assay, a similar result was demonstrated for cancer cells, which showed faster migration with CAF stimulation than control treatment (Supplementary Fig. 2) The spheroid formation assay showed that cancer cells could make larger spheroids under stimulation with CAFs than under control treatment, demonstrating that spheroid formation was enhanced by CM/CAF, and the effects were strong under conditions of direct contact between cancer cells and CAFs (TE4^DC^ and OE19^DC^) (Fig. 2c). However, we did not find any difference between the groups in terms of the number of the cells (data not shown). Therefore, we further investigated EMT and stem-like phenotypic changes that cause these phenomena in cancer cells induced by CAFs and verified markers using flow cytometry and western blotting (WB). First, we observed morphological changes in cancer cells, demonstrating that the population of tumor cells that had fewer cell junctions and elongated pseudopodia was increased in CAF-treated cells compared with untreated control cells. As expected, these changes were observed to be significant in the context of direct contact between cancer cells and CAFs compared to CM/CAF treatment (Fig. 2d). To confirm the influence of direct contact between cancer cells and CAFs, cancer cells were separated from the coculture in vitro and analyzed. The populations of CD44- and CD133-, which are considered markers of cancer stem cells, positive cells were analyzed by flow cytometry. Although there was no difference in the CD44-positive cell population in TE4 or OE19 cells or the CD133-positive population in TE4 cells, the OE19 cell population stimulated with CAFs contained significantly more CD133-positive cells (Fig. 2e). Data on CD44 are not shown. WB demonstrated that E-cadherin expression was decreased and vimentin expression was increased in CAF-stimulated cancer cells by direct contact. These changes were observed to be significant following direct contact between cancer cells and CAFs, as determined by flow cytometry (Fig. 2f). Thus, it is suggested that CAFs induce cancer cells to stem cell-like features and EMT, furthermore these malignant changes may indicate some causes of therapeutic resistance. Furthermore, these results for direct contact in vitro can theoretically support the observed therapeutic resistance of coinoculated tumors, as shown in Fig. 1.

### Reversibility of therapeutic resistance following elimination of CAF stimulation

**Figure 3:**
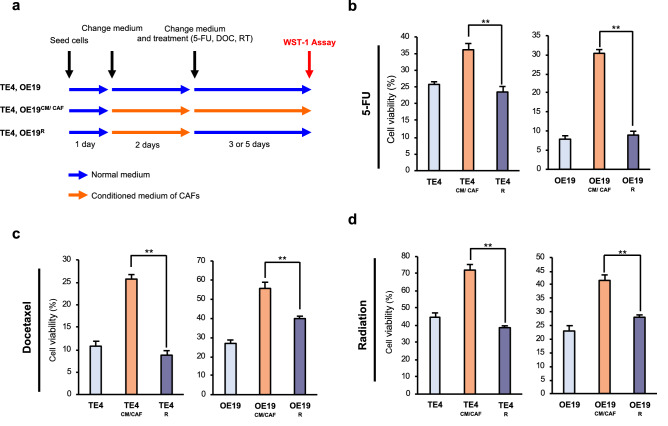
Treatment resistance acquired by CAFs can be improved. (**a**) A schematic of the treatment schedule for this assay is shown. Resistant tumor cells (TE4^CM/CAF^ or OE19^CM/CAF^) were stimulated with conditioned medium from CAFs continuously until analysis. Recovered tumor cells (TE4^R^ or OE19^R^) were stimulated with conditioned medium from CAFs for 2 days, and then this stimulation was blocked by changing the conditioned medium to normal medium (**b**–**d**). Without continuous stimulation by conditioned medium from CAFs, acquired resistance to 5-FU (**b**), docetaxel (**c**) and radiotherapy (**d**) was improved, and cell viability was decreased. (**b**) 5-FU concentration: 500 μM (TE4 and OE19). (**c**) Docetaxel concentration: 10 µM (TE4 and OE19). (**d**) Irradiation dose: 25 Gy (TE4 and OE19). Data are shown as the mean ± SD of three or more independent experiments. Statistical analyses were performed using Dunnett test. **P < 0.01.

In advancing therapeutic strategies targeting CAFs, it is necessary to verify whether resistance to anticancer agents can be improved by remodeling the tumor stroma or targeting CAFs. We assumed that therapeutic resistance acquired by stimulation with CAFs was reversible and might be improved once the influence of CAFs was eliminated. To verify this theory, we constructed a reverse treatment model, as shown in Fig. 3a. In brief, tumor cells were stimulated with CM from CAFs (CM/CAF^TE4, OE19^) continuously until measurement (labeled TE4^CM/CAF^ or OE19^CM/CAF^). On the other hand, in the recovered group, each tumor cell was stimulated with CM from CAFs for 2 days. Then, this CM stimulation was stopped, and the medium was changed back to normal medium (TE4^R^ or OE19^R^) to eliminate CAF effects (Fig. 3a). Although TE4^CM/CAF^ and OE19^CM/CAF^ cells acquired resistance to 5-FU, as in the other experiments, the acquired therapeutic resistance was diminished once stimulation with CM/CAFs was eliminated, as demonstrated in the TE4^R^ and OE19^R^ groups (Fig. 3b). Similar results were found in experiments with docetaxel (Fig. 3c) or radiotherapy (Fig. 3d). In light of these in vitro results, we speculated that CAF elimination could strongly lead to improved therapeutic effects by restructuring the tumor stroma.

### FAP-targeted NIR-PIT leads to CAF-specific rapid cell death

**Figure 4:**
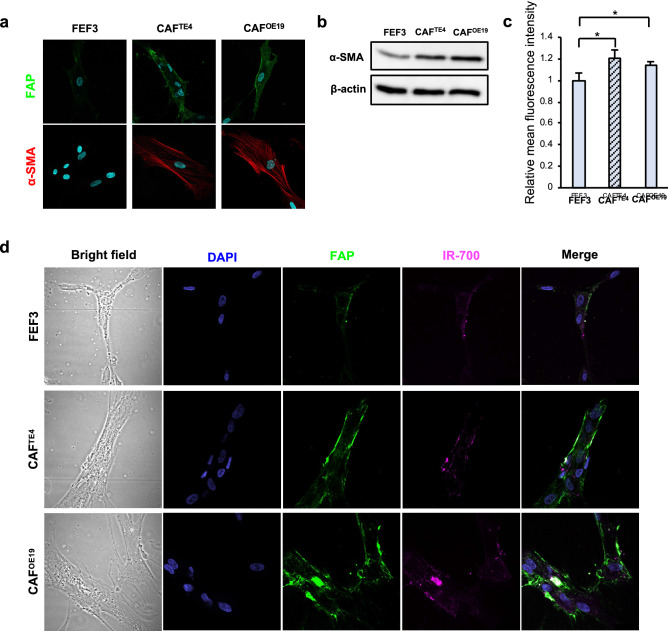
FAP expression in CAFs and conjugation of FAP-IR700. (**a**) Immunofluorescence imaging of fibroblasts is shown. Normal FEF3 cells did not express FAP or αSMA, while CAFs stimulated with tumor cells expressed FAP on the cell surface and αSMA in the cytoplasm. Images are shown at 600 × magnification (green: FAP, red: αSMA, blue: nucleus). (**b**) WB demonstrated that the expression of αSMA was increased in CAFs compared to untreated FEF3 cells. (**c**) Flow cytometry demonstrated the expression of FAP. The relative mean fluorescence intensity of FAP was significantly increased in CAFs compared to untreated FEF3 cells as found by our FlowJo Software. Data are shown as the mean ± SD, Student’s t test. **P < 0.05. (**d**) The binding of FAP on CAFs and FAP-IR700 is shown by immunofluorescence imaging. After 6 h of conjugation with FAP-IR700, fibroblasts were fixed and observed by confocal microscopy. IR700 was observed in the same location as FAP expression in CAFs. However, it was not observed in normal FEF3 cells. Images are shown at 600 × magnification (green: FAP, blue: nucleus, magenta: IR700).

**Figure 5:**
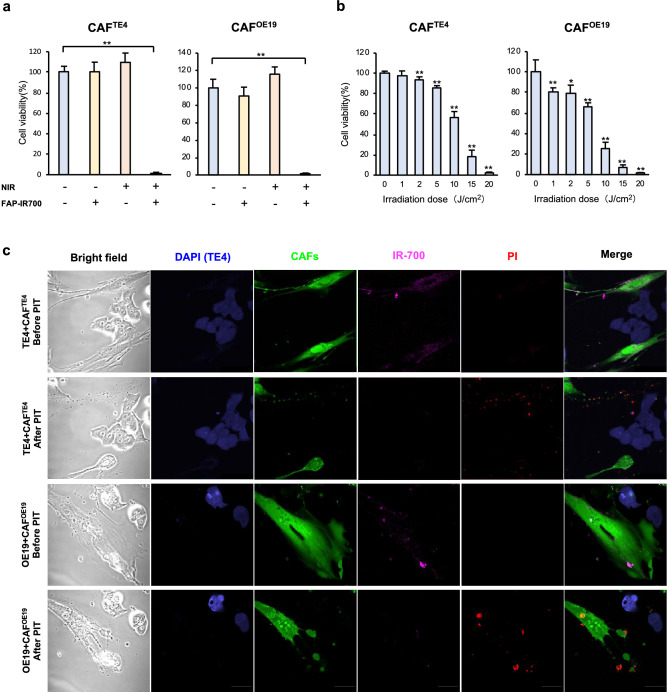
Photoimmunotherapy targeting CAFs expressing FAP induced rapid cell death. (**a**,**b**) A cell viability assay was performed with EC cells treated with NIR-PIT. (**a**) FEF3 were treated with or without 20 J/cm^2^ NIR light irradiation after FAP-IR700 conjugation (20 μg/ml) for 6 h or no conjugation. CAFs were almost dead after conjugation of FAP-IR700 and irradiation. When only FAP-IR700 conjugation or irradiation was performed, CAF viability did not differ significantly. (**b**) NIR-PIT targeting CAFs was evaluated with various irradiation doses of NIR light. The cell viability of CAFs was decreased in an NIR light dose-dependent manner. (**c**) IF imaging shows specific rapid cell death induced by NIR-PIT. After an hour of conjugation with FAP-IR700 (10 μg/ml), cells were irradiated with 5 J/cm^2^ NIR light. PI staining indicated that cell membrane destruction was induced by NIR-PIT. CAFs had bleb formation and were stained with PI. Images were acquired at 600 × magnification (**a**,**b**; data are shown as the mean ± SD, Dunnett test. *P < 0.05, **P < 0.01.).

To verify whether targeted CAF therapy using the NIR-PIT previously reported by us has similar effects on the cancer cells and fibroblasts used in this study, we first confirmed FAP expression in CAFs educated by cancer cells (TE4 and OE19). Although normal fibroblasts expressed FAP mildly, fibroblasts stimulated with cancer medium, CAFs, exhibited strong FAP expression (green) mainly on the cell surface, whereas α-SMA (red) was strongly expressed mainly in the cytoplasm (Fig. 4a). WB showed that the expression of αSMA was increased in CAFs compared to untreated normal fibroblasts and FEF3 cells (Fig. 4b). Similar to the other results, results for flow cytometry analysis demonstrated that the mean fluorescence intensity of FAP was increased in CAFs compared to untreated FEF3 cells (Fig. 4c). Based on these results, we next further confirmed whether FAP-IR700 binds to FAP-expressing cells and evaluated the effect of NIR-PIT on those cells. By fluorescence microscopy, IR-700 (magenta) was observed in the same location as FAP expression on CAFs after conjugation with FAP-IR700, but this was not observed in normal FEF3 cells (Fig. 4d). To verify the effect of FAP-targeted NIR-PIT, FEF3 cells were conjugated with FAP-IR700 (20 μg/ml) or control agents for 6 h. Then, the fibroblasts were treated with or without 20 J/cm^2^ NIR light irradiation. Most of the CAFs educated by either TE4 or OE19 cells exhibited cell disruption (CAF^TE4^: 98.7% reduction, P < 0.01, CAF^OE19^: 98.7%, P < 0.01) by FAP-targeted NIR-PIT. A significant difference was not demonstrated with FAP-IR700 alone or irradiation alone (Fig. 5a). Cell viability was decreased in an NIR light dose-dependent manner (Fig. 5b), demonstrating that such a killing effect on CAFs was increased by strong NIR light. On the other hand, FAP-targeted NIR-PIT did not have any effect on cancer cells in a monoculture, showing specific efficacy for FAP-positive cells (Supplementary Fig. 3). In addition, even in the coculture model in vitro, only CAFs seemed to be damaged by NIR-PIT, which caused cell death. We demonstrated PIT-induced cell membrane destruction in targeted cells by confocal microscopy studies. In these assays, FEF3 cells, so-called CAFs in this case, had bleb formation, shrank and were stained by PI, which indicates the induction of cell death. However, cancer cells that did not express FAP were not affected by FAP-targeted NIR-PIT and were not stained by PI (Fig. 5c). This specificity was supported by morphologic changes using microscopy (Supplementary Fig. 4). These results indicated that we successfully eliminated CAFs specifically with the novel targeting therapy FAP-targeted NIR-PIT.

### Elimination of CAFs improves resistance to chemotherapy

**Figure 6:**
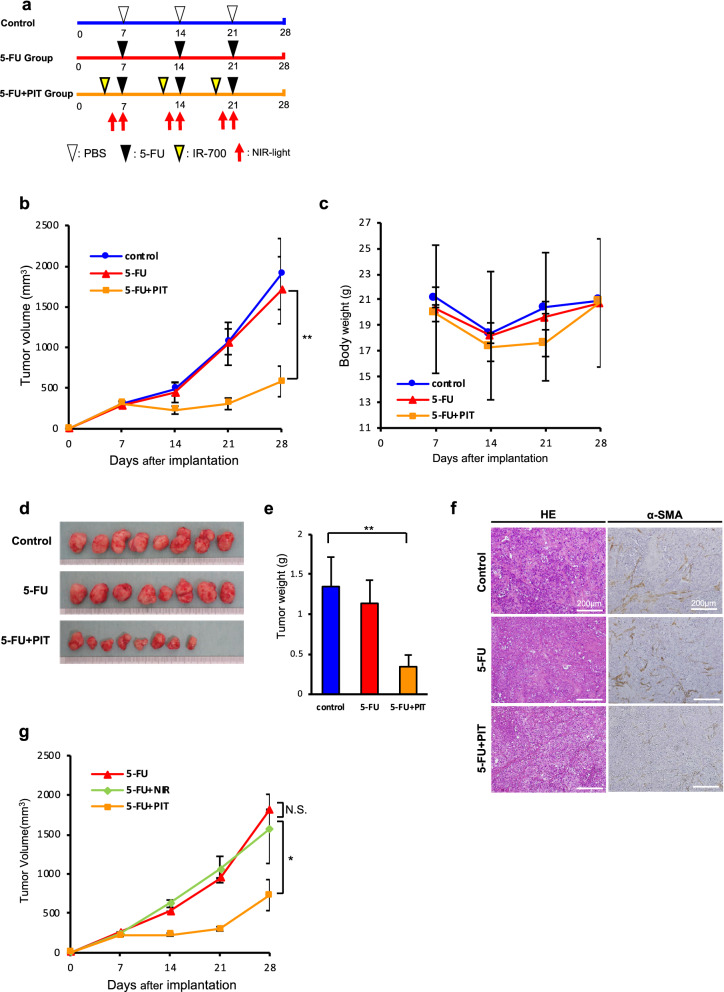
FAP-targeted photoimmunotherapy improved the resistance to chemotherapy induced by CAFs. (**a**) Treatment schedules for the three treatment groups. In each group (n = 8), TE4 cells and CAFs were coinoculated subcutaneously, and the injected mice were randomized into 3 groups (control, 5-FU, and 5-FU plus NIR-PIT). After implantation, tumor growth was monitored from day 7 to day 28. (White arrowhead: PBS injection, black arrowhead: 5-FU intraperitoneal injection (50 mg/kg), yellow arrowhead: FAP-IR700 injection (100 μg/body), red arrow: NIR light irradiation (100 J/cm^2^)). (**b**) Proliferation curves of the 3 groups. Although 5-FU treatment alone could not suppress tumor growth, combination therapy with 5-FU and NIR-PIT could suppress tumor growth. Data are shown as the mean ± SD, Dunnett test. **P < 0.01. (**c**) The mean body weight of each group. Significant differences were not observed among the groups. (**d**) Harvested subcutaneous tumors for each group. (**e**) Tumor weights of the 3 groups. Tumors treated by combination therapy with 5-FU and NIR-PIT showed significantly reduced tumor weights compared with those treated with 5-FU alone. Data are shown as the mean ± SD, Dunnett test. **P < 0.01. (**f**) Immunohistochemistry for αSMA in each resected tumor. Overexpression of αSMA was detected in the control group and 5-FU group, but the expression in the 5-FU + NIR-PIT group was decreased (scale bar, 200 μm). (**g**) The killing effect of NIR light. NIR light could not enhance the effect of 5-FU on tumor growth (5-FU only, n = 4; 5-FU plus NIR light, n = 4; 5-FU plus NIR-PIT, n = 3). Data are shown as the mean ± SD, Dunnett test. *P < 0.05. N.S. indicates no significant difference.

To evaluate the effect of CAFs-PIT and whether the elimination of CAFs by CAFs-PIT overcomes the therapeutic resistance of tumors in vivo, we established CAF coinoculated tumor-bearing mouse models with TE4 cells and compared three groups (the control, 5-FU and 5-FU + NIR-PIT groups). The protocol is shown in Fig. 6a. 5-FU alone could not suppress tumor growth, as shown in Fig. 1e. In marked contrast, combination therapy with 5-FU and NIR-PIT suppressed tumor growth (Fig. 6b, 5-FU: 13.3% reduction, combination of 5-FU and NIR-PIT: 70.9% reduction, day 28, P < 0.01), indicating that elimination of CAFs contributed to the recovery of drug sensitivity and led to tumor suppression. Although it was hypothesized that this method also had some adverse effects, a significant difference in the mean body weight was not found among the groups (Fig. 6c). The reduction in tumor volume mediated by combination therapy with 5-FU and NIR-PIT could clearly be seen macroscopically (Fig. 6d), and the tumors in the combination group demonstrated significantly reduced tumor weights compared with those in the 5-FU treatment alone group (Fig. 6e, 5-FU: 15.6% reduction, combination of 5-FU and NIR-PIT: 74.4% reduction, day 28, P < 0.01). By immunohistochemical analysis, accumulated CAFs were seen in control and untreated NIR-PIT tumors between the tumor cell clusters. However, an emaciated stroma with some fibroblasts was observed in combination-treated tumors (Fig. 6f). Therefore, it was demonstrated that this combination strategy could successfully and safely reverse CAF-induced chemoresistance. Furthermore, to exclude the possibility of some effect of NIR irradiation on nontarget cells, we irradiated CAF coinoculated tumor model mice with NIR light and treated those mice with 5-FU injection; however, no positive effect of NIR irradiation was observed (Fig. 6g).

## Discussion

Novel methods for therapy against cancer cells such as molecular targeted therapies, oncolytic viruses, and NIR-PIT have been developed^38,48,49^; however, there are still many intractable cancers with a poor prognosis, which are called highly malignant cancers. The main types of these tumors are esophageal, pancreatic and scirrhous-type gastric cancers, which are cancer types composed of an abundant organized stroma, and the mechanisms of resistance to conventional treatment for such stroma-rich tumors could be as follows: first is therapeutic resistance acquired via the interaction between cancer and stromal cells, second is a reduction in drug delivery efficiency due to an increase in stromal interstitial pressure, and third is promotion of the secretion of cancer-promoting substances by CAFs during DNA-damaging treatment. For those reasons, novel therapy targeting not only cancer cells but also stromal cells are strongly needed to improve outcomes. However, a definitive treatment targeting the tumor stroma has not yet been established.

In this study, we demonstrated that EC cells originating from squamous cell carcinoma or adenocarcinoma could stimulate fibroblasts into the CAF phenotype, which is the main fibroblast phenotype in the tumor stroma, and furthermore, we suggested that these CAFs influenced cancer cells to develop more malignant phenotypes, which could be related to therapeutic resistance. Although it has already been reported that CAFs originating from tumor tissues exhibit such properties, this is the first report in which the same effects were demonstrated in normal fibroblasts educated by cancer cells. We also showed that the resistance acquired via CAFs could be improved by eliminating CAFs. This reversibility was consistent with the rationale for a therapeutic strategy eliminating CAFs for cancer therapy. In fact, we succeeded in improving the efficacy of chemotherapy by eliminating CAFs by NIR-PIT in vivo*,* while 5-FU alone had poor efficacy in cocultured tumor-bearing mice. Based on our previous reports^43^, our review on day 21 showed a clear difference in tumor reduction at 21 days between NIR-PIT alone and NIR-PIT with 5-FU: 42.5% reduction and 71.4% reduction, respectively, although we did not compare two groups in this research. Therefore, NIR-PIT overcame the resistance of 5-FU, and the therapeutic effect of 5-FU was also added to the NIR-PIT to provide additional tumor suppression. On the other hand, this “CAF targeted therapy” did not affect the cancer cells directly in vitro, as cancer cells did not express FAP (Fig. 5c and Supplementary Fig. 3). According to the observations made after CAFs-PIT, it was confirmed that only cancer cells proliferated in the context of CAF cell death (Supplementary Fig. 4). This suggested that eliminating CAFs may improve resistance to chemotherapy rather than CAFs-targeted NIR-PIT directly affecting cancer cells.

There might be several possibilities to explain why NIR-PIT targeting CAFs improved chemoresistance. First, it has been previously reported that various secreted molecules from CAFs or direct contact between CAFs and cancer cells promotes malignant transformation in cancer cells, leading to the acquisition of EMT markers and a cancer stem-like phenotype, which are known to be factors in chemoresistance; thus, eliminating CAFs may contribute to exhausting this support. Furthermore, Sun et al.^50^ reported that stromal fibroblasts were induced to secrete WNT16B by DNA damage due to anticancer drug administration, which directly attenuated the effects of chemotherapy. Huber et al.^18^ also reported that glial cell line-derived neurotrophic factor (GDNF) was secreted and induced tumor cell proliferation, invasion and resistance to treatment. All of these secretion processes involve paracrine signaling and act on surrounding cancer cells. Therefore, CAF elimination by NIR-PIT prevents elevated cytokine levels, which promote tumor progression even under conventional therapeutic conditions. Second, an increase in the intrastromal pressure within a tumor may cause a decrease in drug delivery efficiency. Although this study did not directly demonstrate a decrease in the interstitial fluid pressure, it has been reported that reducing the number of physical stromal cells in tumors can reduce the interstitial fluid pressure, increasing the deep penetration of antitumor drugs^51^. Furthermore, CAFs secrete extracellular matrix (ECM) components, such as collagen, proteogrican and vascular endothelial growth factor (VEGF); thus, the presence of CAFs causes angiogenesis, which is required for abundant stroma and tumor growth. The effect of NIR-PIT is immediate necrosis of target cells, and rapid loss of CAFs may lead to a decrease in the intrastromal pressure due to the loss of a major stromal component and to suppression of subsequent stromal recomposition by a decrease in VEGF levels. Third, in addition to those reasons, it has been reported that NIR-PIT targeting cancer cells induces perivascular cell death, resulting in massive leakage of nanoparticles into the tumor beds. This phenomenon is called the superenhanced permeation and retention effect (SUPR), and Sano et al.^52^ reported that it was more effective than the normal enhanced permeability and retention (EPR) effect. In this study, we used 5-FU, which is a standard EC treatment, and regression of the tumor stroma by NIR-PIT might induce a perivascular SUPR. In that case, using anticancer nanoparticle preparations may produce even stronger antitumor effects. Considering these possibilities, it seems that the intervention of CAFs-PIT before or simultaneous administration of conventional chemotherapy may have a stronger therapeutic effect.

However, it is known that elimination of ^FAP^CAFs is not easily achieved with previous methods. In a phase II trial with metastatic colorectal cancer patients, the enzymatic activity of FAP could not be inhibited, and no efficacy was not demonstrated, although a humanized anti-FAP antibody (mAb F19; sibrotuzumab) was well tolerated. Some reports have investigated novel strategies to inhibit FAP activity; however, these do not control “cellular CAFs” themselves and may have limited efficacy. Furthermore, since it has been reported that mice with knockdown of FAP-positive stromal cells develop cachexia and anemia, the elimination of all FAP-positive CAFs in the body by systematic administration of some drugs could have significant effects on both normal cells and cells in tumor microenvironments. Therefore, a method that specifically controls only FAP-positive CAFs in tumors would be desirable. From the above, the combination of conventional therapy and FAP-targeted NIR-PIT for CAFs is a reasonable and safe strategy. Furthermore, from the original concept, NIR-PIT can be used with a combination of two types of antibodies conjugated to IR700, and it is speculated that in the future, the effect could be further improved by simultaneously targeting cancer cells and stromal cells.

In conclusion, here, we demonstrated that CAFs-targeted NIR-PIT could work in vivo against EC and overcome therapeutic resistance via CAF elimination. The combined use of novel CAFs-targeted NIR-PIT and conventional anticancer treatments can be expected to provide a more effective and sensible treatment strategy.

## Materials and methods

### Ethics statement

This study was carried out in accordance with the ethical standards of the Helsinki Declaration II and the ethical guidelines for Medical and Health Research Involving Human Subjects^53^. Mouse experiments were performed in a specific pathogen-free environment at the Okayama University animal facility according to institutional guidelines, and all of animal experimental protocols was approved and reviewed by the Ethics Review Committee for Animal Experimentation of Okayama University, Okayama, Japan. All experiments were performed in accordance with all guidelines and regulations indicated by these committees.

### Cell lines and cultures

The human EC cell lines TE4 (HER2-positive squamous cell carcinoma) and OE19 (HER2-positive adenocarcinoma) were used in this study. These cancer cell lines were purchased from the Japanese Collection of Research Bioresources (JCRB) Cell Bank (Osaka, Japan). Primary human fetal esophageal fibroblasts (FEF3 cells) were isolated from the human fetal esophagus, and GFP-FEF3 cells were stably transduced with the gene for green fluorescent protein (GFP) as described previously^36,47^. All experiments were performed with mycoplasma-free cells. All cell lines were authenticated by the JCRB Cell Bank (National Institute of Biomedical Innovation, Osaka, Japan) using short tandem repeat analysis. Cells were cultured in Dulbecco’s modified Eagle’s medium (DMEM; Sigma-Aldrich, St. Louis, MO) supplemented with 10% fetal bovine serum (FBS; Gibco, Thermo Fisher Scientific, Waltham, MA) and 1% penicillin/streptomycin (Sigma-Aldrich, St Louis, MO) in a humidified incubator at 37 °C with 5% CO_2_.

### Antibodies and reagents

The following antibodies were used in this study: a polyclonal anti-α-SMA antibody (Abcam, Cambridge, UK) for western blotting (WB) and immunofluorescence (IF); an anti-α-SMA mAb (Sigma-Aldrich, St Louis, MO) for immunohistochemistry (IHC); an anti-FAP mAb (Abnova, Taipei, Taiwan) for WB; an anti-FAP mAb (R&D Systems, Minneapolis, MN) for flow cytometry, IF, and IR700-conjugated procedures; an anti-E-cadherin mAb (Cell Signaling Technology, Danvers, MA) for WB; an anti-N-cadherin mAb (Cell Signaling Technology) for WB; an anti-Vimentin mAb (Cell Signaling Technology) for WB; a FITC-conjugated anti-CD44 mAb (Miltenyi Biotec GmbH, Bergisch Gladbach, Germany) for flow cytometry; an APC-conjugated anti-CD133/2 mAb (Miltenyi Biotec GmbH) for flow cytometry; and an anti-β-actin antibody (Sigma-Aldrich) for WB.

### Treatment of FEF3 cells with conditioned medium

For preparation of conditioned medium (CM), tumor cells were cultured (in T175 flasks) with DMEM containing 10% FBS. After 24 h, the medium was replaced with DMEM containing 2% FBS, and the cells were incubated. After 2 days, the supernatants were harvested, centrifuged at 1000 rpm for 5 min, and collected. The CM from tumor cells (CM/TE4 or CM/OE19) was stored at − 30 °C until use. FEF3 cells were cultured (in T175 flasks) with DMEM containing 10% FBS. After 24 h, the medium was replaced with DMEM containing 2% FBS or CM/TE4 or CM/OE19, and the cells were incubated for 2 days. These fibroblasts were termed normal FEF3, CAF^TE4^ or CAF^OE19^, and the CMs were termed CM/NF, CM/CAF^TE4^ and CM/CAF^OE19^, respectively. Supernatants were harvested, centrifuged at 1000 rpm for 5 min and collected as CMs described above. The CM from CAFs was stored at − 30 °C until use.

### Stimulation of tumor cells

Tumor cells were cultured by three different methods. Tumor cells were cultured with DMEM containing 10% FBS. After 24 h, the medium was replaced with DMEM containing 2% FBS (control) or CM from activated FEF3 cells (CM/CAF^TE4^ or CM/CAF^OE19^); these tumor cells were termed TE4^CM/CAF^ or OE19^CM/CAF^. Cells were stimulated for 4 days. Alternatively, tumor cells and FEF3 cells were mixed and cultured with DMEM containing 10% FBS. After 24 h, the medium was replaced with CM/CAF^TE4^ or CM/CAF^OE19^; these tumor cells were termed TE4^Direct Contact^ (TE4^DC^) or OE19^DC^. These cells were stimulated for 4 days and observed with a microscope (Olympus, Tokyo, Japan).

### Flow cytometry

Tumor cells were stimulated for 4 days with CM/CAF^TE4^ or CM/CAF^OE19^ (TE4^CM^ or OE19^CM^) or cocultured with tumor cells and fibroblasts (TE4^DC^ or OE19^DC^). In the coculture model, tumor cells mixed with FEF3 cells were isolated using anti-ErbB-2 magnetic microbeads (Miltenyi Biotec GmbH, Bergisch Gladbach, Germany). The cells were centrifuged at 300×*g* for 10 min. The cell pellets were then resuspended in 300 μL of buffer containing a final concentration of 0.5% FBS and 2 mM ethylenediaminetetraacetic acid dissolved in calcium- and magnesium-free phosphate-buffered saline (pH 7.2) and incubated with 100 μL of FcR Blocking Reagent (Miltenyi Biotec GmbH, Bergisch Gladbach, Germany) and 100 μL of human anti-ErbB-2 microbead-conjugated antibodies for 30 min. The cells were then separated using a MiniMACS cell separator (Miltenyi Biotec GmbH, Bergisch Gladbach, Germany). This procedure was repeated three times to improve purity. The expression of CD44 (Miltenyi Biotec GmbH) and CD133 (Miltenyi Biotec GmbH) was analyzed using a fluorescence-activated cell sorter (FACScan, Becton Dickinson, Franklin Lakes, NJ) with FlowJo software (TreeStar, Ashland, OR). FEF3 cells were stimulated with CM from tumor cells (CM/TE4 or CM/OE19) for 2 days. Then, these cells (CAF^TE4^ or CAF^OE19^) were labeled with a primary mouse anti-FAP antibody (R&D, MAB3715) and FITC-conjugated anti-mouse secondary antibody. The expression of FAP was analyzed using a fluorescence-activated cell sorter.

### Cell viability assay following chemotherapy or radiotherapy

TE4 and OE19 cells were plated in 96-well microplates (TE4: 3 × 10^3^/well, OE19: 5 × 10^3^/well) and incubated (at 37 °C with 5% CO_2_) for 24 h. Then, the medium was changed to DMEM supplemented with 2% FBS (control), CM from CAFs (CM/CAF^TE4^ or CM/CAF^OE19^) or normal FEF3 cells (CM/NF), and the cells were cultured for 2 days. The tumor cells cultured with CM/NF were termed TE4^CM/NF^ or OE19^CM/NF^. After stimulation by culture for 2 days, the tumor cells were treated with the indicated concentrations of 5-fluorouracil (FU) and docetaxel or doses of radiotherapy. Cell proliferation was measured by using water-soluble tetrazolium-1 (WST-1) assays (Rosch Diagnostics GmbH, Mannheim, Germany) 3 days after chemotherapy or 5 days after irradiation. WST-1 reagent (10 μL) was added to 100 μL of cell suspension and incubated for 4 h. The absorbance in wells was measured with a microplate reader set at a wavelength of 450 nm with a reference wavelength of 690 nm.

### Migration and invasion assays

For migration assays, 24-well cell culture inserts with a pore size of 8.0 μm (Falcon, Corning, Corning, NY) were prepared. In the migration assay, tumor cells (1 × 10^5^) were seeded in the upper compartment in 500 μL of serum-free DMEM or CM from CAFs (CM/CAF^TE4^ or CM/CAF^OE19^) or FEF3 cells (CM/NF). The bottom well was filled with 750 μL of DMEM containing 10% FBS. After incubation (at 37 °C with 5% CO_2_) for 24 h, the cells on the upper surface of the transwell insert were removed using cotton swabs. The migrated cells on the lower surface were stained with crystal violet and observed under a microscope at 200 × magnification. In invasion assays, we used transwell inserts coated with Matrigel (Corning), and the subsequent procedures were the same as those for the migration assays. Images were captured in 5 different fields, and the number of cells was then counted visually.

### Scratch assay

Tumor cells and FEF3 cells were cultured to 95% confluence in 6-well plates. A wound was made by scratching a line across the bottom of the plate through the confluent cell monolayer using a 200-μL pipette tip. Then, the medium was changed to normal medium (control) or CM from CAFs (CM/CAF^TE4^ or CM/CAF^OE19^), and the cells were cultured for 2 days. Migratory cells were observed under a microscope (Olympus, Tokyo, Japan).

### Sphere formation assay

Basement Membrane Matrix Matrigel (Corning, Corning, NY) was placed in 96-well round-bottom microplates (20 μL/well) and solidified (at 37 °C with 5% CO_2_) for 30 min. Tumor cells (1 × 10^4/^/well) and FEF3 cells (5 × 10^3^/well) were plated onto the 3D Matrigel and cultured in 2% Matrigel in DMEM supplemented with 2% FBS or CM from CAFs (CM/CAF^TE4^ or CM/CAF^OE19^) for 3 days. Spheroids were observed under a microscope at 100 × magnification. Spheroid size was analyzed with ImageJ software (http://rsb.info.nih.gov/ij/) ^54^.

### Western blot analysis

Tumor cells were stimulated for 4 days with CM/CAF^TE4^ or CM/CAF^OE19^ (TE4^CM/CAF^ or OE19^CM/CAF^) or cocultured with fibroblasts (TE4^DC^ or OE19^DC^). In the coculture model, the tumor cells mixed with FEF3 cells were isolated using the same method as that used for flow cytometry. FEF3 cells were stimulated with CM from tumor cells (CM/TE4 or CM/OE19) for 2 days (CAF^TE4^ or CAF^OE19^). Primary antibodies against E-cadherin (Cell Signaling Technology), N-cadherin (Cell Signaling Technology), Vimentin (Cell Signaling Technology), αSMA (Abcam), and β-actin (Sigma) were used. Cells were washed, lysed in SDS buffer, and centrifuged. The supernatants were collected and subjected to WB. Proteins were electrophoretically transferred to Hybond-polyvinylidene difluoride transfer membranes (GE Healthcare Life Science) and incubated with a primary antibody, followed by peroxidase-linked secondary antibodies (Amersham Bioscience). An ECL Prime Western Blotting Detection Reagent (GE Healthcare UK Ltd.) was used to detect the peroxidase activity of the bound antibody.

### Immunofluorescence staining for FAP and α-SMA in FEF3 cells

FEF3 cells (5 × 10^3^/well) were seeded in 4-well chambered slides. After 24 h, the supernatants were replaced with normal medium or CM from tumor cells (CM/TE4 or CM/OE19), and the cells were cultured for 2 days. After stimulation, the cells were fixed with 4% paraformaldehyde in PBS for 15 min and blocked with 3% bovine serum albumin for 30 min (for FAP staining) or cold 100% methanol for 30 min on ice (for αSMA staining). The slides were incubated with a primary antibody for an hour (FAP; R&D, MAB3715) or overnight (αSMA; Abcam) on ice. After washing twice with PBS, the slides were incubated with the appropriate secondary antibody, FITC-conjugated anti-mouse IgG or Alexa 568-conjugated goat anti-mouse IgG (Invitrogen), for an hour on ice. The slides were further stained with 4′,6-diamidino-2-phenylindole (DAPI) and mounted by using Fluorescent Mounting Medium (Dako Cytomation). Then, the cells were analyzed with a confocal laser microscope (FV10i, Olympus, Tokyo, Japan).

### Synthesis of FAP-IR700

An anti-FAP antibody (1 mg, R&D, MAB3715) was incubated with IR700 (63.5 μg, 32.5 nmol) in 0.3 mol/L Na2HPO4 (pH 8.5) at room temperature (RT) for 2 h in the dark. The mixture was purified with a Sephadex G50 column (PD-10; GE Healthcare). The protein concentration was determined with a Bio-Rad protein assay kit (Bio-Rad, CA) by measuring the absorption at 280 nm for FAP mAb and 689 nm for IR700 with spectroscopy. With this sample, the number of fluorophore molecules per FAP mAb was adjusted to approximately 2 (Supplementary Fig. 5). This conjugated antibody was defined as FAP-IR700.

### Immunofluorescence staining of FAP-IR700-conjugated FEF3 cells

FEF3 cells (5 × 10^3^/well) were seeded on 4-well chambered slides. After 24 h, the supernatants were replaced with normal medium or CM from tumor cells (CM/TE4 or CM/OE19), and the cells were cultured for 2 days. After stimulation, FAP-IR700 was added to the culture medium at 20 μg/mL and incubated for 6 h at 37 °C. Then, the cells were washed with PBS and fixed with cold 4% paraformaldehyde for 15 min on ice. After washing twice with PBS, the slides were incubated with a FITC-conjugated secondary antibody for an hour on ice. The slides were further stained with DAPI. Then, the cells were analyzed with an FV10i confocal laser microscope. FAP-IR700 could be detected by IR700 fluorescence with a 590- to 650-nm excitation filter and a 665- to 740-nm bandpass emission filter and FITC fluorescence.

### Cell viability assay for NIR-PIT

Tumor cells (TE4: 1 × 10^4^/well, OE19: 6 × 10^3^/well) were plated (in 96-well microplates) and stimulated with CM from CAFs (CM/CAF^TE4^ or CM/CAF^OE19^) or normal medium for 2 days. FEF3 cells (2 × 10^3^/well) were plated (in 96-well microplates) and stimulated with CM from tumor cells (CM/TE4 or CM/OE19) for 4 days. After stimulation, FAP-IR700 was added to the culture medium at 20 μg/mL and incubated for 6 h at 37 °C. After washing cells with PBS, the medium was replaced with normal medium, and NIR light was administrated to the cells with a red light-emitting diode (LED) at 20 J/cm^2^ or the indicated intensity (L700-05AU 700 nm, Epitex Co, Kyoto, Japan) with a power density of 15 mW/cm^2^ as measured using an optical power meter (PM 100, Thorlabs, Inc., Newton, NJ). After exposure to NIR light, cell proliferation was immediately measured by using WST-1 assays, as described above.

### Fluorescence microscopy

To detect fibroblast-specific cell death, fluorescence microscopy was performed. Tumor cells (5 × 10^3^/well, stained with the CellTracker Blue CMAC dye, 1:400, Invitrogen, Waltham, MA) and GFP-FEF3 cells (2.5 × 10^3^/well) were plated on 8-well chambered slides and stimulated with CM from tumor cells (CM/TE4 or CM/OE19) for 2 days, as described above. After stimulation, FAP-IR700 was added to the culture medium at 10 μg/mL and incubated for an hour at 37 °C. After conjugation, the medium was replaced with normal medium, and NIR light was administrated to the cells with a red LED at 5 J/cm^2^. After 30 min of irradiation, propidium iodide (PI) was added to the medium at an approximate final concentration of 1 µg/mL and incubated at 37 °C for 30 min. The cells were observed with an FV10i confocal laser microscope.

### Xenograft tumor model and experiment

All animal studies were approved by the Ethics Review Committee for Animal Experimentation of Okayama University, Okayama, Japan and followed the ARRIVE guidelines for reporting animal research^55^. BALB/c athymic mice (BALB/c-nu/nu) were purchased from Clea (Tokyo, Japan). Six-week-old female mice were used to establish a xenograft tumor model. A total of 3 × 10^6^ TE4 cells and 1.5 × 10^6^ CAF^TE4^ cells (stimulated for 4 days, as described above) were suspended in a 50% mixture of Matrigel (Corning, Corning, NY) and inoculated subcutaneously into the right flank.

To evaluate the resistance to chemotherapy, we generated 2 models, mice inoculated with TE4 cells alone and mice coinoculated with TE4 and CAF^TE4^ cells (Fig. 1d,e). We randomized the mice used for both models into 2 groups (n = 4/group) after inoculation, and 50 mg/kg 5-FU or PBS was injected intraperitoneally on days 7, 11, and 15. Tumor growth was monitored from day 7 to day 28. To investigate the efficacy of NIR-PIT, mice coinoculated with TE4 and CAF^TE4^ cells were randomized into 3 groups (n = 8/group) after inoculation for the following treatments: (1) PBS treatment (control); (2) 5-FU treatment; and (3) 5-FU and NIR-PIT treatment (Fig. 6a–c). Five days after cell inoculation, 100 μg/body of FAP-IR700 was injected intraperitoneally, and NIR light was administered at 100 J/cm^2^ on days 1 and 2 after injection. According to the previous results demonstrated that two NIR light doses kill up to 80% of target- expressing cells, based on the biodistribution of IR700 was high in 24 h after APC injection and decreased over the following days^56,57^. Irradiation was performed by using an LED light source (L690-66-60 with Lens550; EPITEX, Inc.) with 690 nm as the peak wavelength with a power density of 25 mW/cm^2^ as measured using an optical power meter same as in vitro. 50 mg/kg 5-FU or PBS was injected intraperitoneally once a week on the same date as the second irradiation with NIR light. Each treatment was performed for three cycles. To eliminate the antitumor effect of NIR light alone on tumors (Fig. 6g), mice coinoculated with TE4 and CAF^TE4^ cells were randomized into 3 groups (n = 3 or 4/group) after inoculation for the following treatments: (1) 5-FU treatment; (2) 5-FU and NIR light treatment; and (3) 5-FU and NIR-PIT treatment. Five days after cell inoculation, 100 μg/body of FAP-IR700 was injected intraperitoneally^58^. NIR light was administered at 100 J/cm^2^ by an LED light source on days 1 and 2 after injection. 50 mg/kg 5-FU was injected intraperitoneally once a week on the same date as the second irradiation with NIR light. Each treatment was performed for three cycles. Tumor volume and body weight were measured once a week. Tumor volumes were estimated using the following formula: V = 1/2 × L x W^2^, where V is the tumor volume, L is the length, and W is the width.

### Immunohistochemical staining

Resected tumors were fixed in 10% paraformaldehyde and embedded in paraffin. Paraffin sections were immunohistochemically stained. An anti-α-SMA antibody (Sigma) was used for immunohistochemical staining. TE4 and CAF^TE4^ tumors were harvested for histological analysis after treatment. Sections on microslides were deparaffinized with xylene, hydrated using a diluted alcohol series, and immersed in H_2_O_2_ in methanol to quench endogenous peroxidase activity. The sections were treated with a citrate buffer solution. To reduce nonspecific staining, each section was blocked with Serum-Free Protein Block (Dako, Agilent Technologies, Santa Clara, CA) for 15 min. The sections were then incubated at RT with an anti-SMA antibody (Sigma) diluted in Dako REAL Antibody Diluent (Dako) for 30 min, followed by three washes with buffer. The sections were then incubated with an anti-mouse antibody (EnVision + System/HRP, Mouse (DAB +), Dako) for 30 min at RT. The chromogen used was DAB plus liquid (Dako). The sections were counterstained with Meyer’s hematoxylin, as reported previously^7,47,59^.

### Statistical analysis

All statistical analyses were performed with SPSS advanced statistical software (IBM, Chicago, IL, USA). Data are shown as the mean ± standard deviation (SD). A comparison of continuous variables between two groups for in vitro and in vivo assays was performed using a two-sided Student’s t test. For multiple-group comparison, Dunnett test was used. Differences between groups were considered to be statistically significant when the P value was * < 0.05 or ** < 0.01.

### Consent for publication

All authors read and approved the manuscript. All contributing authors approved the submission of this version of the manuscript and asserted that the document represents valid work. No contributing authors have any disclosures to make.

## Supplementary Information

Supplementary Information

## Data Availability

The datasets generated and/or analyzed during this study are included in this published article (and its Supplementary files), otherwise available from the corresponding author upon reasonable request.

## Abbreviations

APC: Antibody-photosensitizer conjugate
CAF: Cancer-associated fibroblast
CM: Conditioned medium
DMEM: Dulbecco’s modified eagle medium
EC: Esophageal carcinoma
ECM: Extracellular matrix
EMT: Epithelial mesenchymal transition
EPR: Enhanced permeability and retention
FACS: Fluorescence-activated cell sorting
FAP: Fibroblast activation protein
FBS: Fetal bovine serum
FEF3: Fetal esophageal fibroblasts
FSP1: Fibroblast-specific protein 1
5-FU: 5-Fluoreuracil
GFP: Green fluorescent protein
ICD: Immunogenic cell death
IL-6: Interleukin-6
mAB: Monoclonal antibody
NIR: Near infrared
PBS: Phosphate-buffered saline
PDGFR: Platelet-derived growth factor receptor
PIT: Photoimmunotherapy
RT: Room temperature
SEM: Standard error of the mean
SMA: Smooth muscle actin
SUPR: Superenhanced permeation and retention effect
TGF: Transforming growth factor
VEGF: Vascular endothelial growth factor
WB: Western blotting
XTT: Sodium 2,3,-bis(2-methoxy-4-nitro-5-sulfophenyl)-5-[(phenylamino)-carbonyl]-2H-tetrazolium
