# Supplementary material for: Fibroblast activation protein targeted near infrared photoimmunotherapy (NIR PIT) overcomes therapeutic resistance in human esophageal cancer
Source: Sci Rep. 2021 Jan 18;11:1693. doi: 10.1038/s41598-021-81465-4 (PMC7814141; doi:10.1038/s41598-021-81465-4)
Supplement: Supplementary file 1 — Supplementary Information [file 41598_2021_81465_MOESM1_ESM.doc]

**Fibroblast activation protein targeted near infrared photoimmunotherapy (NIR PIT) overcomes therapeutic resistance in human esophageal cancer**

Ryoichi Katsube^1^, Kazuhiro Noma^1^, Toshiaki Ohara^1,2^, Noriyuki Nishiwaki^1^, Teruki Kobayashi^1^, Satoshi Komoto^1^, Hiroaki Sato^1^, Hajime Kashima^1^, Takuya Kato^1^,

Satoru Kikuchi^1^, Hiroshi Tazawa^1,3^, Shunsuke Kagawa^1^, Yasuhiro Shirakawa^1^,

Hisataka Kobayashi^4^, Toshiyoshi Fujiwara^1^

^1^Department of Gastroenterological Surgery, Okayama University Graduate School of Medicine, Dentistry and Pharmaceutical Sciences, Okayama, Japan

^2^Department of Pathology & Experimental Medicine, Okayama University Graduate School of Medicine, Dentistry and Pharmaceutical Sciences, Okayama, Japan

^3^ Center for Innovative Clinical Medicine, Okayama University Hospital, Okayama, Japan

^4^Molecular Imaging Program, Center for Cancer Research, National Cancer Institute, US National Institutes of Health, Bethesda, Maryland, USA

**Supplementary Fig. 1. Normal fibroblasts could not induce resistance to therapy in cancer cells.**

After stimulation with CM from CAFs (TE4^CM/CAF^ or OE19^CM/CAF^) or normal FEF3 (TE4^CM/NF^ or OE19^CM/NF^) for 2 days, tumor cells were treated with the indicated concentrations of 5-FU and docetaxel, and cell viability was measured 3 days later. Tumor cells were irradiated with the indicated dose of radiotherapy, and cell viability was measured 5 days later. There was a significant difference in the viability of cells stimulated with CAF CM or FEF3 cell CM after treatment. FEF3 cells could not induce resistance to therapy. 5-FU concentration: TE4: 500 μM, OE19: 500 μM. Docetaxel concentration: TE4: 10 μM, OE19: 10 μM. Irradiation dose: TE4: 25 Gray, OE19: 25 Gray. Statistical analyses were performed using the Student t test. **P* < 0.01.

**Supplementary Fig. 2. The migratory activity of cancer cells was enhanced in the presence of CAFs.**

Two days after scratching, migratory cells were observed. Tumor cells stimulated with CAFs exhibited enhanced migratory activity. In addition, it was confirmed that tumor cells migrated following CAFs in the presence of CAFs. (Scale bar, 500 μm).

**Supplementary Fig. 3. FAP-targeted photoimmunotherapy did not induce cancer cell death.**

Cancer cells were treated with or without 20 J/cm^2^ NIR light irradiation and 6 hours of FAP-IR700 conjugation (10 μg/ml). Regardless of exposure to FAP-IR700 conjugation or irradiation, cell viability did not differ significantly.

**Supplementary Fig. 4. FAP-targeted photoimmunotherapy had high selectivity.**

After an hour of conjugation with FAP-IR700 (10 μg/ml), cells were irradiated with 5 J/cm^2^ NIR light. After 2 days, the proliferation of cancer cells was not suppressed, although CAFs almost disappeared, and their proliferation was not observed. White arrowhead: lost CAFs after treatment (Scale bar, 200 μm).


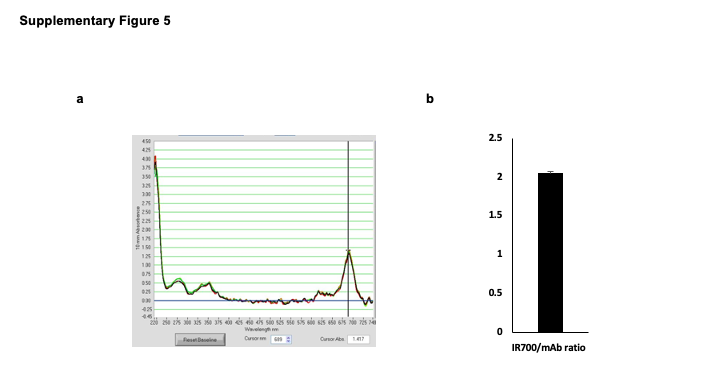


**Supplementary Fig. 5. Conjugation rate of IR700 to anti-FAP antibody.**

(a) The protein concentration was determined with a Bio-Rad protein assay kit (Bio-Rad, CA) by measuring the absorption at 280nm for FAP mAb and 689nm for IR700 with spectroscopy. (b) The number of fluorophore molecules per FAP mAb was adjusted to approximately 2.


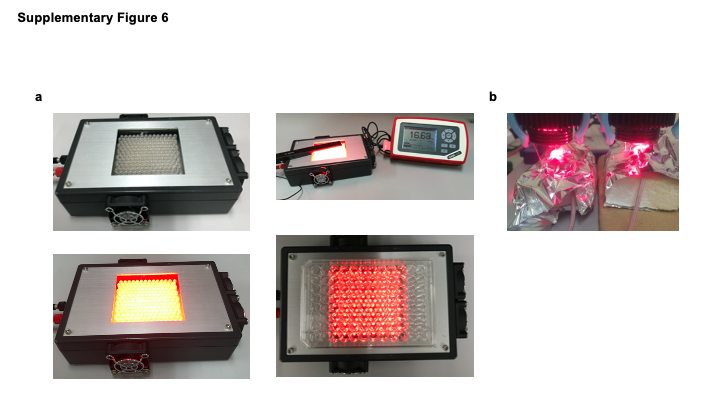


**Supplementary Fig. 6. The LED system used in this study.**

(a) NIR-PIT was performed with a red LED as measured using an optical power meter *in vitro experiment*. NIR light was administrated well by well. (b) The red LED used *in vivo* experiment.
